# Assessment of the FTO gene polymorphisms (rs1421085, rs17817449 and rs9939609) in exercise-trained men and women: the effects of a 4-week hypocaloric diet

**DOI:** 10.1186/s12970-019-0307-6

**Published:** 2019-09-02

**Authors:** Jose Antonio, Sarah Knafo, Madaline Kenyon, Alina Ali, Cassandra Carson, Anya Ellerbroek, Cailey Weaver, Justin Roberts, Corey A. Peacock, Jaime L. Tartar

**Affiliations:** 1 Exercise and Sport Science, Nova Southeastern University, 3401 South University Drive, Davie, FL 33328 USA; 2 Department of Psychology and Neuroscience, NSU Florida, Davie, FL USA; 3 Cambridge Centre for Sport & Exercise Sciences, Anglia Ruskin University, Cambridge, UK

**Keywords:** Body composition, Fat mass, Cortisol, Genotype

## Abstract

**Background:**

Variations in the fat mass and obesity-associated gene (FTO) are associated with obesity; however, it is unclear if changes in energy intake affect the adaptive response to caloric restriction in those with risk variants. The three FTO single nucleotide polymorphisms (SNPs), rs1421085, rs17817449 and rs9939609, are in strong linkage disequilibrium. Thus, the purpose of this investigation was to determine the role of these FTO SNPs vis-à-vis the effects of a 4-week hypocaloric diet on body composition in exercise-trained men and women. Two salivary biomarkers that associate with energy expenditure were also assessed (cortisol and salivary alpha-amylase, sAA).

**Methods:**

Forty-seven exercise-trained men (*n* = 11) and women (*n* = 36) (mean ± SD: age 32 ± 9 years; height 169 ± 8 cm, body mass index 24.5 ± 2.9 kg/m^2^, hours of aerobic training per week 4.9 ± 3.8, hours of weight training per week 3.9 ± 2.4, years of training experience 13.4 ± 7.0) completed a 4-week hypocaloric diet (i.e., decrease total calories by ~ 20–25% while maintaining a protein intake of ~ 2.0 g/kg/d). Subjects were instructed to maintain the same training regimen and to decrease energy intake via carbohydrate and/or fat restriction during the treatment period. Body composition was assessed via dual-energy X-ray absorptiometry (DXA) (Model: Hologic Horizon W; Hologic Inc., Danbury CT USA). Total body water was determined via a multifrequency bioelectrical impedance (BIA) device (InBody 770). Saliva samples were collected pre and post intervention in order to genotype the participants as well as to determine the concentrations of cortisol and sAA.

**Results:**

Of the 47 subjects, 15 were of normal risk for obesity whereas 32 were carriers of the FTO gene risk alleles. Subjects were grouped based on their genotype for the three FTO SNPs (i.e., rs1421085, rs17817449 and rs9939609) due to their strong linkage disequilibrium. We have classified those with the normal obesity risk as “non-risk allele” versus those that carry the “risk allele” (i.e., both heterozygous and homozygous). Both groups experienced a significant decrease in total energy intake (*p* < 0.01); non-risk allele: pre kcal 2081 ± 618, post kcal 1703 ± 495; risk allele: pre kcal 1886 ± 515, post kcal 1502 ± 366). Both groups lost a significant amount of body weight (*p* < 0.01); however, there was no difference between groups for the change (post minus pre) in each group (risk allele change: − 1.0 ± 1.2 kg, non-risk allele change: − 1.2 ± 1.4 kg). Additionally, both groups lost a significant amount of fat mass (*p* < 0.01) with no differences between groups for the change in fat mass (risk allele change for fat mass: 1.1 ± 0.7 kg, non-risk allele change − 0.9 ± 0.4 kg). There were no significant changes in either group for fat free mass or total body water. The change in salivary alpha-amylase or cortisol was not different between groups.

**Conclusions:**

In the short-term (i.e., 4 weeks), exercise-trained men and women consuming a hypocaloric diet that is relatively high in protein experience similar changes in body composition due exclusively to a decrement in fat mass and independent of FTO allele status. Therefore, weight and fat loss on a hypocaloric diet is, at least in the short-term, unaffected by the FTO gene.

## Background

There are several single nucleotide polymorphisms (SNPs) on the fat mass and obesity-associated (FTO) gene that are associated with obesity in adults and children [1–7]. For the FTO SNP rs8050136, homozygotes for the risk allele (A/A) have 1.67 times greater risk of obesity than those who do not have the allele [8]. Furthermore, the FTO genotype was associated with changes in body composition as a result of regular exercise. Thus, carriers of the C allele showed three times greater fat mass and percent body fat losses than the A/A homozygotes [8]. It is unclear if the FTO genotype has an interaction with macronutrient intake. One group tested the effects of an FTO variant (rs1558902) on weight loss in response to 2-year diet interventions. This study demonstrated that a high-protein diet might be beneficial for weight loss and improvement of body composition and fat distribution in individuals with the risk allele of the FTO variant rs1558902 [9]. In an investigation that was comprised of 788 unrelated Mexican-Mestizo individuals, the FTO SNPs (rs9939609, rs1421085, and rs17817449) were a major risk factor for obesity [10]. Recent data characterized the FTO SNP (rs1421085), which results in a T to C nucleotide substitution, in a cohort of exercise-trained men and women [11]. The study population of 108 exercise-trained individuals included professional mixed martial arts fighters, competitive distance runners, collegiate swimmers, stand-up paddlers as well as a cohort of recreational bodybuilders. The data showed that C allele carriers had significantly higher fat mass and percent body fat relative to the TT group. However, due to the cross-sectional nature of this investigation, it was not possible to determine causality regarding the FTO gene and body composition [11]. Nonetheless, it is evident that investigations of the FTO gene focus primarily on overweight or obese individuals [3, 5, 12, 13]. The current investigation is a follow up on the cross-sectional data previously obtained from a cohort of exercise-trained individuals [11]. Certainly, the cross-sectional data shows that the presence of the risk variant for the FTO gene is associated with an elevated fat mass. However, one cannot assign causality based on observational data. Thus, the purpose of this investigation was to determine if body composition was differentially altered in a group of exercise-trained men and women based on their FTO genotype. This is the first investigation in exercise-trained men and women that have examine the role of the FTO SNPs as it relates to caloric restriction and body composition assessment. The FTO SNPs (rs9939609, rs1421085, and rs17817449) were analyzed due to their strong linkage disequilibrium (i.e., there is a non-random association of allele types in these SNPs).

## Methods

### Participants

Subjects came to the laboratory on two occasions for body composition assessment and to provide saliva samples. In accordance with the Helsinki Declaration, the University’s Institutional Review Board approved all procedures that involved human subjects. Written informed consent was obtained prior to participation. In order to control for the circadian influence on cortisol, all testing took place between 1130 and 1400. Participants were instructed not to exercise, eat, or drink anything other than water 3 h prior to testing. For the duration of the 4-week treatment period, subjects were instructed as well to not alter their training regimen substantially.

### Body composition

Subjects had their body composition assessed via dual-energy X-ray absorptiometry (DXA) (Model: Hologic Horizon W; Hologic Inc., Danbury CT USA). Quality control calibration procedures were performed on a spine phantom. Subjects wore typical athletic clothing and removed all metal jewelry. They were positioned supine on the DXA within the borders delineated by the scanning table. Each whole body scan took approximately 7 min. In addition, total body water was determined via bioelectrical impedance (Model: InBody 770, Cerritos, California USA). Subjects stood on the platform of the device barefoot with the soles of their feet on the electrodes. Subjects then grasped the handles of the unit with their thumb and fingers to maintain direct contact with the electrodes. They stood still for ~ 1 min while maintaining their elbows fully extended and their shoulder joint abducted to about a 30 degree angle.

### Food diary

Subjects kept a diary (i.e., ~3 days per week) of their food intake via a smartphone app (MyFitnessPal®). The use of mobile apps for dietary self-reporting has been previously reported [14]. Every subject had previously used this mobile app. The MyFitnessPal® app is a database comprised of over 5 million foods that have been provided by users via entering data manually or by scanning the bar code on packaged goods. Thus, the data themselves are primarily derived from food labels (i.e., Nutrition Facts Panel) derived from the USDA National Nutrient database. Subjects were instructed to decrease their food intake by ~ 20–25% while maintaining a relatively high protein intake (~ 2 g per kilogram body weight daily). Thus, subjects decreased their carbohydrate and fat intake to promote a energy deficit.

### Genotyping

Genomic DNA was extracted in a QIAcube instrument following the manufacturer’s standard protocol for saliva nucleic acid extraction (QIAGEN, Valencia, CA). After isolation, allelic discrimination for the three FTO SNPs was determined via real-time polymerase chain reaction (PCR) using TaqMan SNP genotyping assays using fluorogenic probes (Applied Biosystems, CA) with the following primer sequences.

rs1421085:

TAGCAGTTCAGGTCCTAAGGCATGA**[C/T]**ATTGATTAAGTGTCTGATGAGAATT, rs17817449: GTGTTTCAGCTTGGCACACAGAAAC**[G/T]**GTTTTAATTTAACAGTCCAGCTCCT, rs9939609: GGTTCCTTGCGACTGCTGTGAATTT**[A/T]**GTGATGCACTTGGATAGTCTCTGTT.

For all three genotyping assays, thermal cycling was performed on StepOne Real-Time PCR system (Applied Biosystems, CA). The amplification mix contained the following ingredients: 12.5 μL of PCR master mix (QIAGEN, Valencia, CA), 1.25 μL of TaqMan 20X working stock, 10.25 μL of RNase- and DNase-free water (Sigma), and 1.0 μL of sample DNA, in a total volume of 25 μL per single tube reaction. The PCR conditions were 95 °C for 10 min followed by 40 repeated cycles of 95 °C for 15 s and 60 °C for 60 s. Genotypes were determined automatically via the StepOne software (Applied Biosystems, CA) based on the fluorescence signals. Samples were run in duplicate and in the case of a call discrepancy, samples were rerun.

It is known that the three FTO SNPs that we examined (i.e., rs1421085, rs17817449 and rs9939609) are in strong linkage disequilibrium. Each SNP is briefly described in Table 3 [1, 11, 15, 16]. Thus, we have classified those with the normal obesity risk as “non-risk allele” versus those that carry the “risk allele” (i.e., both heterozygous and homozygous).

### Salivary cortisol and alpha-amylase

At each testing session the participants provided saliva samples for sAA and cortisol quantification. An additional saliva sample was collected at baseline for genotyping. Saliva was collected from each participant by unstimulated passive salivate. Immediately after collection, sample tubes were stored in a − 20 °C freezer, and later quantified via human enzyme immunoassay kits per the manufacturer’s instructions (Salimetrics LLC, USA).

#### Cortisol

Saliva samples were run in duplicate and quantified via a human cortisol enzyme immunoassay (EIA) kit per the manufacturer’s instructions (Salimetrics LLC, USA). The samples were immediately read in a BioTek ELx800 plate reader (BioTek Instruments, Inc., USA) at 450 nm with a correction at 630 nm. All samples were within the detection ranges indicated in the cortisol immunoassay kit, and the variations of sample readings were within the expected limits. Final concentrations for the biomarkers were generated by interpolation from the standard curve in μg/dL.

#### Salivary alpha amylase (sAA)

Saliva samples were run in duplicate and quantified via a human Kinetic Enzyme Assay Kit per the manufacturer’s instructions (Salimetrics LLC, USA). The samples were immediately read in a BioTek ELx800 plate reader (BioTek Instruments, Inc., USA) at 405 nm. All samples were within the detection ranges indicated in the assay kit, and the variations of sample readings were within the expected limits. Final concentrations for the biomarkers were generated via absorptivity over 2min and generated in U/mL of activity.

### Statistical analysis

All data are presented as the mean ± SD (standard deviation). A series of paired (pre vs post) and unpaired (delta score between groups) t-tests were used to assess the relationship between FTO genotype (risk versus non-risk allele) and body composition, diet, and salivary biomarkers. The distribution of allele frequencies was determined by the Hardy–Weinberg Exact (HWE) test, and the association of allele status was analyzed using the chi-square test. All reported *p*-values are two-tailed with a priori significance level of *p* < 0.05. (GraphPad Prism 6).

## Results

Of the 73 initial subjects that volunteered for the investigation, 10 dropped out (i.e., did not show up for post-testing) and 16 were non-compliant (i.e., did not decrease energy intake). Of the 47 compliant subjects, 15 were of normal risk for obesity whereas 32 were carriers of the risk alleles for the FTO gene (Tables 1, 2 and 3). The HWE test for rs17817449 was χ2 = 0.03, *p* = 0.86 and rs9939609 was χ2 = 0.81, *p* = 0.37, suggesting that the population is consistent with Hardy-Weinberg Equilibrium. The HWE test for rs1421085 was χ2 = 4.02, *p* = 0.04. This was due to a higher number of observed vs. expected heterozygotes (29 vs. 22). Subjects were grouped based on their genotype. Both groups decreased total energy intake significantly (~ 400 kcal) with no difference between groups (Table 4). The decrease in energy intake was due to a significant decrease in carbohydrate (~ 70 g) and fat consumption (~ 20 g); however, total protein intake did not change (Table 4).

**Table 1:** Genotype

SNP	Genotype	Description
rs1421085	TT	Normal risk
	C/−	In exercise-trained men and women, those carrying the risk C allele had a significantly higher fat mass and % body fat [11]. This SNP is most likely risk variant showing the highest association with obesity [1, 11].
rs17817449	TT	Normal risk
	G/−	This SNP is associated with body mass index, weight and waist circumference [16].
rs9939609	TT	Normal risk
	A/−	This SNP is associated with diminished satiety [16]. Those who are AT or AA consumed between 125 and 280 kcal more each day than those with the protective TT genotype [7]; however, energy expenditure is not affected.

**Table 2:** Physical characteristics and training history

	Risk Allele (*n* = 32), 7 males	Non-Risk Allele (*n* = 15), 4 males
Age (years)	34 ± 9	28 ± 9
Height (centimeters)	167 ± 7	173 ± 9
Body Weight (kilograms)	68.5 ± 12.5	73.9 ± 12.8
Body Mass Index (kilograms/meter^2^)	24 ± 3	24 ± 3
Hours of Aerobic Training Per Week	5.2 ± 4.5	4.8 ± 3.0
Hours of Resistance Training Per Week	4.2 ± 2.8	3.9 ± 1.0
Total Number of Years Training	14.7 ± 8.1	11.8 ± 4.5

Data are expressed as the mean ± SD. There were no differences between groups. If an individual carried the risk alleles for at least two of the three SNPs (i.e., rs1421085, rs17817449, rs9939609) they were classified as part of the Risk Allele group

**Table 3:** Baseline characteristics according to FTO genotype for the three SNPs

FTO SNP	Genotype	Age (yr)	Sex n (m/f)	Height (cm)	Body Weight (kg)	Body Fat %
rs1421085	TT	28 ± 10	6/8	174 ± 8	76.1 ± 11.1	26.7 ± 7.2
	CT + CC	34 ± 9	6/27	167 ± 7	67.7 ± 12.5	26.4 ± 5.8
rs17817499	TT	28 ± 10	5/11	173 ± 9	73.6 ± 12.8	26.2 ± 7.4
	GT + GG	34 ± 8	6/25	167 ± 7	68.4 ± 12.6	26.8 ± 5.7
rs9939609	TT	27 ± 10	4/10	173 ± 9	73.8 ± 12.7	27.1 ± 7.0
	AT + AA	34 ± 9	8/25	168 ± 7	68.7 ± 12.4	26.2 ± 5.9

Data are expressed as the mean ± SD. Legend: *cm* Centimeters, *f* Female, *kg* Kilograms, *m* Male, *yr* Years of age. Note: we grouped heterozygotes/homozygotes that were at increased risk for obesity for all three SNPs (i.e., C/−, G/− and A/−)

**Table 4:** Nutrition

	Risk Allele	Non-Risk Allele
Pre-kcal	1868 ± 477	2081 ± 618
Post-kcal	1465 ± 346*	1703 ± 495*
Change (kcal)	− 403 ± 280	− 378 ± 308
Pre-kcal/kg/d	27.4 ± 6.3	28.6 ± 8.9
Post-kcal/kg/d	21.7 ± 4.1*	23.8 ± 7.5*
Change (kcal/kg/d)	−5.7 ± 4.0	−4.9 ± 4.4
Pre-carbohydrate (grams)	190 ± 60	227 ± 88
Post-carbohydrate (grams)	129 ± 58*	154 ± 72*
Change (grams)	−67 ± 50	−74 ± 42
Pre-carbohydrate (g/kg/d)	2.8 ± 0.8	3.2 ± 1.4
Post-carbohydrate (g/kg/d)	1.8 ± 0.6*	2.2 ± 1.2
Change (g/kg/d)	−1.0 ± 0.7	−1.0 ± 0.6
Pre-protein (grams)	130 ± 51	131 ± 42
Post-protein (grams)	142 ± 41^#^	143 ± 48
Change (grams)	12 ± 30	12 ± 44
Pre-protein (g/kg/d)	1.9 ± 0.7	1.8 ± 0.5
Post-protein (g/kg/d)	2.1 ± 0.6^#^	2.0 ± 0.6
Change (g/kg/d)	0.2 ± 0.4	0.2 ± 0.6
Pre-fat (grams)	67 ± 24	72 ± 23
Post-fat (grams)	47 ± 15*	58 ± 20*
Change (grams)	−21 ± 14	−15 ± 11
Pre-fat (g/kg/d)	1.0 ± 0.3	1.0 ± 0.3
Post-fat (g/kg/d)	0.7 ± 0.2*	0.8 ± 0.2*
Change (g/kg/d)	−0.3 ± 0.2	−0.2 ± 0.2

Date are mean ± SD. Legend – *d* Day, *g* Grams, *kcal* Kilocalories, *kg* Kilograms

*p < 0.01 Post versus Pre

^#^*p* < 0.05 Post versus Pre

There were no differences between groups for the change

**Fig. 1:**
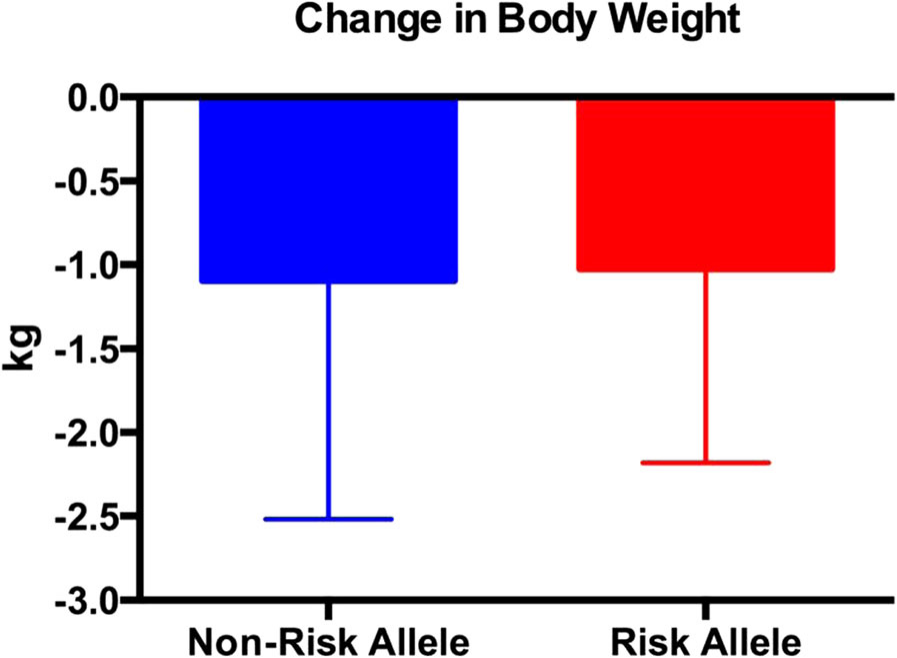
The change in body weight (post weight minus pre weight). There were no between-group differences. Data are expressed as the mean ± SD

**Fig. 2:**
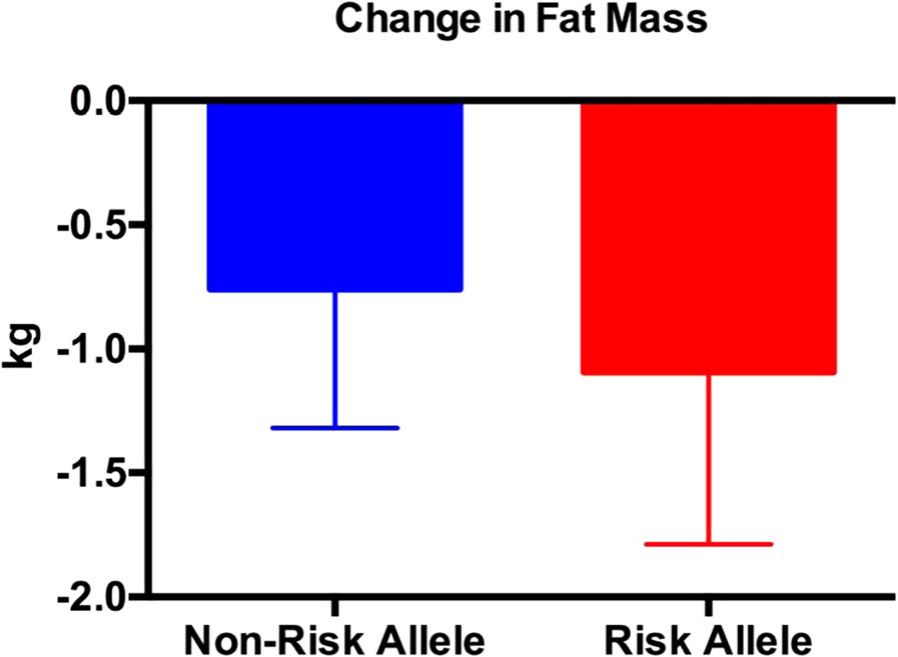
The change in fat mass (post fat mass minus pre fat mass). There were no between-group differences. Data are expressed as the mean ± SD

**Fig. 3:**
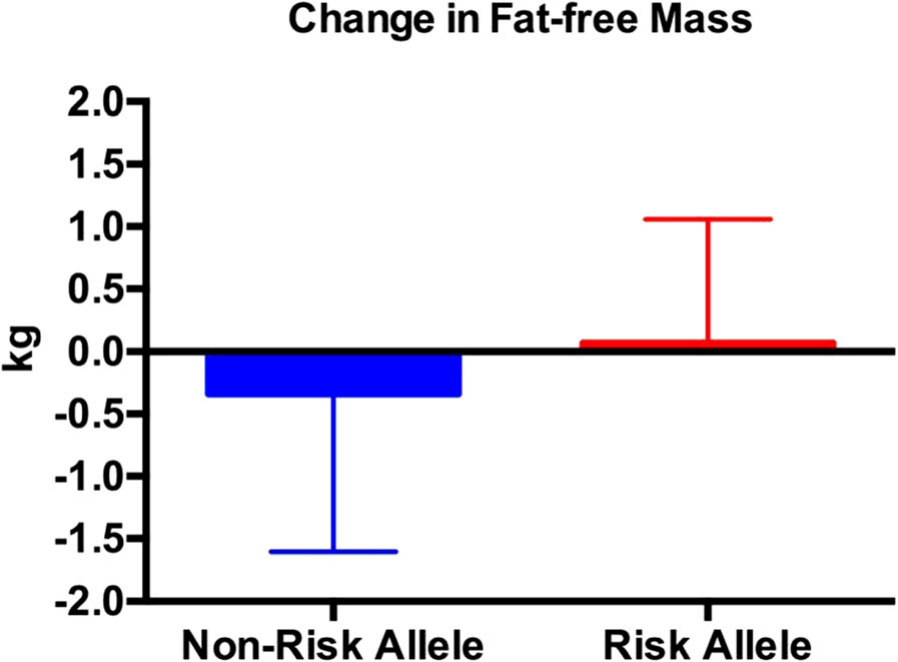
The change in fat-free mass (post fat-free mass minus pre fat-free mass). Fat-free mass did not change in either group. There were no between-group differences. Data are expressed as the mean ± SD

**Fig. 4:**
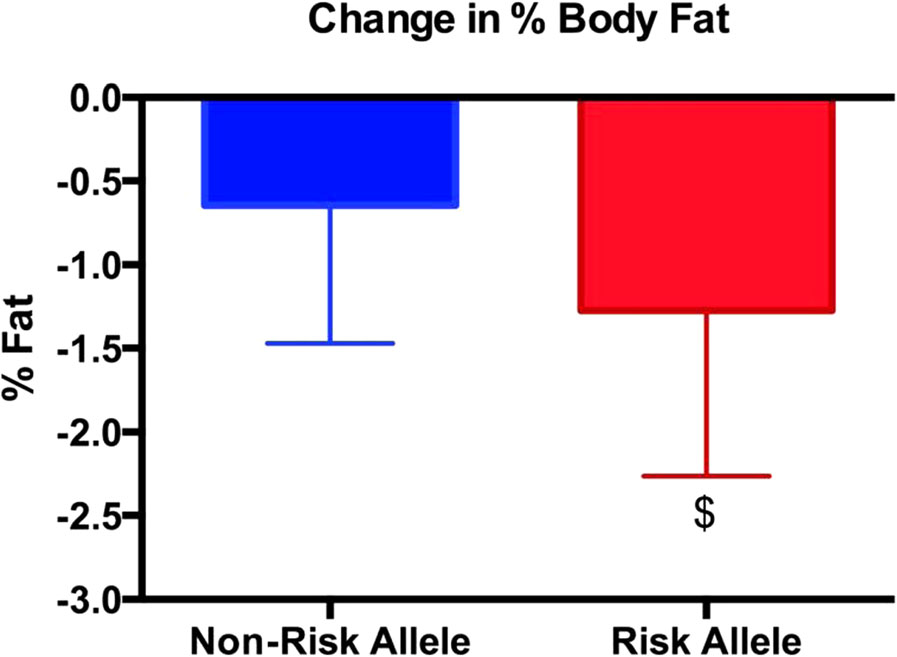
The change in percent body fat (post % body fat minus pre % body fat). The Risk Allele group experienced a greater change in % body fat versus the Non-Risk Allele group (*p* < 0.05). Data are expressed as the mean ± SD

**Fig. 5:**
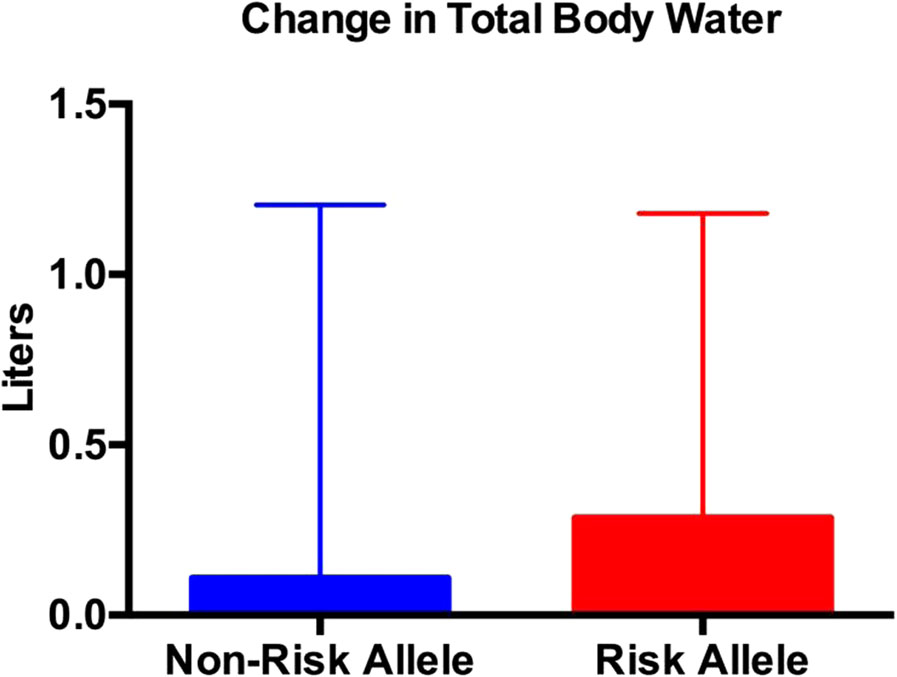
The change in total body water (post total body water minus pre total body water). There weas no change in total body water. There were no between-group differences. Data are expressed as the mean ± SD

Both the risk and non-risk allele groups experienced a significant decrease in body weight, fat mass, % body fat, trunk fat mass, and lower extremity fat mass (Tables 5 and 6); however, only the risk allele group experienced a significant decline in upper extremity fat mass (Table 6). There were no differences between groups for any of the body composition measures (Figs. 1, 2, 3 and 4; Tables 5 and 6) except percent body fat (Fig. 5).

**Table 5:** Body composition

	Risk Allele	Non-Risk Allele
Pre-body weight (kg)	68.7 ± 12.6	73.6 ± 12.8
Post-body weight (kg)	67.7 ± 12.5*	72.4 ± 12.3*
Change (kg)	−1.0 ± 1.2	− 1.2 ± 1.4
Pre-fat free mass (kg)	47.8 ± 9.2	51.5 ± 10.7
Post-fat free mass (kg)	47.9 ± 9.1	51.2 ± 10.4
Change (kg)	0.1 ± 1.0	− 0.3 ± 1.3
Pre-fat mass (kg)	18.2 ± 6.1	19.3 ± 6.1
Post-fat mass (kg)	17.1 ± 6.1*	18.4 ± 6.0*
Change (kg)	−1.1 ± 0.7	−0.9 ± 0.4
Pre-body fat %	26.5 ± 5.8	26.2 ± 7.4
Post-body fat %	25.2 ± 5.8*	25.4 ± 7.5*
Change	−1.3 ± 1.0**	−0.8 ± 0.7
Pre-bone mineral content (kg)	2.6 ± 0.5	2.9 ± 0.5
Post-bone mineral content (kg)	2.6 ± 0.5	2.9 ± 0.5
Change (kg)	0.0 ± 0.1	0.0 ± 0.1
Pre-total body water (liters)	38.8 ± 7.8	42.2 ± 8.4
Post-total body water (liters)	39.1 ± 7.6	42.1 ± 8.1
Change (liters)	0.3 ± 0.9	−0.1 ± 1.0

Data are expressed as the mean ± SD. Legend: *kg* Kilogram

*p < 0.01 post versus pre within groups. There were no between-group differences except for the change in % body fat.**

**Table 6:** Segmental fat mass changes

	Risk Allele	Non-Risk Allele
Pre-trunk fat mass (kg)	7.6 ± 2.9	8.0 ± 3.2
Post-trunk fat mass (kg)	6.8 ± 2.7*	7.5 ± 3.1*
Change (kg)	−0.7 ± 0.6	− 0.5 ± 0.3
Pre-upper extremity fat mass (kg)	2.1 ± 0.8	2.4 ± 0.8
Post-upper extremity fat mass (kg)	2.0 ± 0.7*	2.3 ± 0.9
Change (kg)	−0.1 ± 0.3	−0.1 ± 0.3
Pre-lower extremity fat mass (kg)	7.3 ± 3.2	7.8 ± 2.3
Post-lower extremity fat mass (kg)	7.1 ± 3.3^#^	7.6 ± 2.4^#^
Change (kg)	−0.2 ± 0.3	−0.2 ± 0.4

Date are expressed as the mean ± SD. Legend: *kg* Kilogram

*p < 0.01 post versus pre

#*p* < 0.05 post versus pre

There were no differences between groups for the change

There was a significant increase in sAA, a marker of sympathetic nervous system (SNS) activity, in the risk allele group [17]; however, there were no between-group differences for the change in sAA. Moreover, there were no significant changes in salivary cortisol (Table 7).

**Table 7:** Biomarkers

	Risk Allele	Non-Risk Allele
Pre-sAA (U/mL)	90.7 ± 71.8	98.2 ± 77.1
Post-sAA (U/mL)	132.0 ± 116.4^#^	113.3 ± 67.5
Change (U/mL)	42.1 ± 104.4	15.0 ± 63.3
Pre-cortisol (micrograms/dL)	0.259 ± 0.181	0.221 ± 0.134
Post-cortisol (micrograms/dL)	0.163 ± 0.181	0.213 ± 0.182
Change (micrograms/dL)	−0.097 ± 0.275	− 0.008 ± 0.158

Data are mean ± SD. Legend: *dL* Deciliter, *mL* Milliliter, *sAA* Salivary alpha-amylase

^#^*p* < 0.05 Post versus Pre. There were no between group differences for the change in sAA or cortisol

## Discussion

To the authors’ knowledge, this is the first randomized controlled trial on the FTO gene that has examined the effect of caloric restriction on a group of normal weight men and women with several years of regular exercise training. The data from the current investigation demonstrates that the restriction of dietary energy from carbohydrate and fat result in a similar loss of fat mass in the risk versus non-risk allele groups. Interestingly, the change in percent body fat was significantly greater in the risk allele (− 1.3%) versus non-risk allele (− 0.8%) group. Though not significantly different, the risk allele group lost 1.1 kg of fat mass compared to 0.9 kg in the non-risk allele group. Both groups lost most of their fat mass from the trunk followed by the lower and upper extremities. In essence, it is clear that short-term energy restriction is effective regardless of whether one carries the ns for the three FTO SNPs that we examined. Although not significant, the risk allele group trended towards greater alterations in body composition.

The current investigation is a follow-up to the cross-sectional study conducted on 108 highly trained individuals that included professional mixed martial arts fighters, competitive distance runners, collegiate swimmers, stand-up paddlers, and recreational bodybuilders. In that prior investigation, it was found that C allele carriers of the FTO SNP rs1421085 had significantly higher fat mass and percent body fat versus the TT group. Interestingly, cortisol levels were significantly higher in the TT group relative to the C allele carriers thus demonstrating that resting cortisol is unrelated to body fat mass [11]. In a large-scale cross-sectional study of 846 healthy Finnish males of Caucasian origin, subjects were genotyped for the FTO SNP rs8050136. The AA genotype of the FTO SNP rs8050136 is associated with a higher BMI and greater waist circumference compared to the genotype CC. It was discovered that there was no relationship between the FTO gene and aerobic or neuromuscular exercise performance. Thus, aerobic fitness may not modify the effect of FTO variation on body composition traits [18]. Another group investigated whether body composition and metabolism were modulated by the FTO SNP rs9939609 in a group of young women engaged in exercise training. A group of 201 young Polish women were examined before and after the completion of a 12-week training program. Subjects with AA and AT genotypes had higher BMI during the entire study period compared with the TT genotype. Although BMI, basal metabolic rate, percent body fat, fat mass, fat-free mass, total body water, high-density lipoprotein, and glucose changed significantly during the training program, there were no differences between groups (i.e., those with the risk alleles, AA and AT, responded similarly as those that were the TT genotype) [19]. This further demonstrates that given the same exercise intervention, the adaptive response is similar between risk and non-risk allele groups.

According to Harbron et al. the risk alleles of the FTO polymorphisms were associated with poorer eating behaviors (e.g., higher hunger, internal locus for hunger, and emotional disinhibition scores), as well as a higher intake of high fat foods and refined starches and more depressive symptoms [4]. It has been suggested that specific macronutrient diet composition can influence the adaptive response as it relates to the FTO gene. Thus, it would seem plausible that dietary habits can modify the FTO gene risk allele influence on obesity [20]. For instance, in adolescents whose fat intake was below 30%, the A allele of rs9939609 was not associated with adiposity. Conversely, adolescents whose fat intake was between 30 and 35% of energy, the rs9939609 SNP was associated with a 1.9% higher body fat per risk allele and in those whose fat intake was higher than 35%, it was associated with a 2.8% higher body fat per risk allele. At least with this specific FTO SNP rs9939609, adiposity may be exacerbated in adolescents consuming high fat diets [21]. The current investigation showed that a decrease in energy consumption (~ 5–6 kcal/kg/day decrease) resulted in a fat mass loss with no change in fat-free mass. Our subjects consumed a relatively high protein diet (~ 2 g/kg/d) during the 4-week treatment. It is likely that the maintenance of fat-free mass during caloric restriction was due both to the high protein intake as well as the ~ 9 h per week of exercise training this cohort of subjects performed. Di Renzo et al. studied a group of 188 Italian subjects to determine the effects of a Mediterranean diet on body composition during a 4-week treatment. The FTO SNP rs9939609 was determined in this cohort. They discovered that although the diet reduced fat mass, the FTO genotype had no influence on the loss of fat mass [22]. Hubacek et al. analyzed the FTO SNP rs17817449 in 6024 adults aged 45–69 years to assess the role of diet and physical activity. This FTO variant was significantly associated with body mass index and basal metabolic rate; on the other hand, it was not associated with physical activity, total energy intake or with energy intakes from fat, carbohydrates, proteins or alcohol [23]. Zhang et al. conducted the most extensive study to date on the influence of a dietary intervention in obese adults and the FTO SNP rs1558902 [9]. The FTO SNP rs1558902 was genotyped in 742 obese adults who were randomly assigned to one of four diets differing in the proportions of fat, protein, and carbohydrate: the target percentages of energy derived from fat, protein, and carbohydrate in the four diets were 20, 15, and 65%; 20, 25, and 55%; 40, 15, and 45%; and 40, 25, and 35%. Body composition and fat distribution were measured by dual-energy x-ray absorptiometry and computed tomography. They discovered that carriers of the rs15589002 risk allele (A) had a greater reduction in weight, body composition, and fat distribution in response to a high-protein diet, whereas an opposite genetic effect was observed on changes in fat distribution in response to a low-protein diet. This data suggest that a higher protein diet may be beneficial for body composition those with the risk allele of the FTO variant rs1558902 [9]. Certainly at this point, it is unclear how diet and the FTO gene interact. However, based on the limited number of randomized controlled trials, it is evident that caloric restriction can produce a loss of fat mass regardless of one’s FTO genotype.

An association between an FTO SNP and cortisol levels was previously reported in a cross-sectional study of exercise-trained individuals where the non-risk allele group had higher cortisol levels [11]. Additionally, there is an association between high cortisol levels and obesity [24]. However, the current investigation found no change in cortisol (post minus pre) in either group. Also, despite the fact that sAA increased in the risk allele group, the differences between the groups (change in sAA) were no significantly different. Thus, it is not clear what relationship cortisol or sAA has with the FTO gene and changes in body composition in exercise-trained individuals.

### Limitations and future directions

Although we did not find any differences in body composition changes between subjects that were of normal risk versus those that carried the risk alleles for the FTO gene, it should be noted that our study was fairly short-term (i.e., 4 weeks) and a longer treatment duration may have resulted in a different outcome. Moreover, it would be intriguing to assess whether protein overfeeding would result in a different body composition response in those that carry the risk variants.

## Conclusion

Exercise-trained men and women that carry the risk alleles for the FTO SNPs (rs1421085, rs17817449 and rs9939609) had similar changes in body composition compared to those at normal risk after a 4-week period of energy restriction. The decrease in percent body fat was due exclusively to a loss of fat mass. Total body water did not change in either group; thus, we can rule out a decrease in percent body fat due to an elevation of total body water. At least in the short-term, individuals can lose fat mass in spite of their FTO genotype. Thus one can conclude that changes in body composition are unaffected by the FTO gene (i.e., energy restriction will produce a loss of fat mass whether one has the risk or non-risk alleles).

## Data Availability

The author should be contacted for data requests.

## Abbreviations

DXA: Dual energy x-ray absorptiometry
EIA: Enzyme immunoassay
FTO gene: Fat mass and obesity associated gene
Kg: Kilogram
MMA: Mixed martial arts
Nucleotides A, T, C, G stand for: Adenine, thymine, cytosine, guanine
sAA: Salivary alpha amylase
SNP: Single nucleotide polymorphism

## References

[1] Dina C, Meyre D, Gallina S, Durand E, Korner A, Jacobson P, Carlsson LM, Kiess W, Vatin V, Lecoeur C (2007). Variation in FTO contributes to childhood obesity and severe adult obesity. Nat Genet.

[2] Chauhan G, Tabassum R, Mahajan A, Dwivedi OP, Mahendran Y, Kaur I, Nigam S, Dubey H, Varma B, Madhu SV (2011). Common variants of FTO and the risk of obesity and type 2 diabetes in Indians. J Hum Genet.

[3] Chuenta W, Phonrat B, Tungtrongchitr A, Limwongse C, Chongviriyaphan N, Santiprabhob J, Tungtrongchitr R (2015). Common variations in the FTO gene and obesity in Thais: a family-based study. Gene.

[4] Harbron J, van der Merwe L, Zaahl MG, Kotze MJ, Senekal M (2014). Fat mass and obesity-associated (FTO) gene polymorphisms are associated with physical activity, food intake, eating behaviors, psychological health, and modeled change in body mass index in overweight/obese Caucasian adults. Nutrients.

[5] Liu C, Mou S, Cai Y (2013). FTO gene variant and risk of overweight and obesity among children and adolescents: a systematic review and meta-analysis. PLoS One.

[6] Price RA, Li WD, Zhao H (2008). FTO gene SNPs associated with extreme obesity in cases, controls and extremely discordant sister pairs. BMC Med Genet.

[7] Speakman JR, Rance KA, Johnstone AM (2008). Polymorphisms of the FTO gene are associated with variation in energy intake, but not energy expenditure. Obesity.

[8] Rankinen T, Rice T, Teran-Garcia M, Rao DC, Bouchard C (2010). FTO genotype is associated with exercise training-induced changes in body composition. Obesity.

[9] Zhang X, Qi Q, Zhang C, Smith SR, Hu FB, Sacks FM, Bray GA, Qi L (2012). FTO genotype and 2-year change in body composition and fat distribution in response to weight-loss diets: the POUNDS LOST trial. Diabetes.

[10] Villalobos-Comparan M, Teresa Flores-Dorantes M, Teresa Villarreal-Molina M, Rodriguez-Cruz M, Garcia-Ulloa AC, Robles L, Huertas-Vazquez A, Saucedo-Villarreal N, Lopez-Alarcon M, Sanchez-Munoz F (2008). The FTO gene is associated with adulthood obesity in the Mexican population. Obesity.

[11] Antonio J, Knafo S, Kapoor R, Tartar JL (2018). A fat mass and obesity-associated gene polymorphism influences fat mass in exercise-trained individuals. J Int Soc Sports Nutr.

[12] Zhang Q, Xia X, Fang S, Yuan X (2018). Relationship between fat mass and obesity-associated (FTO) gene polymorphisms with obesity and metabolic syndrome in ethnic Mongolians. Med Sci Monit.

[13] Srivastava A, Mittal B, Prakash J, Srivastava P, Srivastava N, Srivastava N (2016). Association of FTO and IRX3 genetic variants to obesity risk in North India. Ann Hum Biol.

[14] Turner-McGrievy GM, Beets MW, Moore JB, Kaczynski AT, Barr-Anderson DJ, Tate DF (2013). Comparison of traditional versus mobile app self-monitoring of physical activity and dietary intake among overweight adults participating in an mHealth weight loss program. J Am Med Inform Assoc : JAMIA.

[15] Speakman JR (2015). The ‘Fat mass and obesity Related’ (FTO) gene: mechanisms of impact on obesity and energy balance. Curr Obes Rep.

[16] Do R, Bailey SD, Desbiens K, Belisle A, Montpetit A, Bouchard C, Perusse L, Vohl MC, Engert JC (2008). Genetic variants of FTO influence adiposity, insulin sensitivity, leptin levels, and resting metabolic rate in the Quebec family study. Diabetes.

[17] Arhakis A, Karagiannis V, Kalfas S (2013). Salivary alpha-amylase activity and salivary flow rate in young adults. Open Dent J.

[18] Huuskonen A, Lappalainen J, Oksala N, Santtila M, Hakkinen K, Kyrolainen H, Atalay M (2012). Aerobic fitness does not modify the effect of FTO variation on body composition traits. PLoS One.

[19] Leonska-Duniec A, Jastrzebski Z, Zarebska A, Maciejewska A, Ficek K, Cieszczyk P (2018). Assessing effect of interaction between the FTO a/T polymorphism (rs9939609) and physical activity on obesity-related traits. J Sport Health Sci.

[20] Przeliorz-Pyszczek A, Regulska-Ilow B (2017). The role of macronutrient intake in reducing the risk of obesity and overweight among carriers of different polymorphisms of FTO gene. A review. Rocz Panstw Zakl Hig.

[21] Labayen I, Ruiz JR, Huybrechts I, Ortega FB, Arenaza L, Gonzalez-Gross M, Widhalm K, Molnar D, Manios Y, DeHenauw S (2016). Dietary fat intake modifies the influence of the FTO rs9939609 polymorphism on adiposity in adolescents: the HELENA cross-sectional study. Nutr Metab Cardiovasc Dis: NMCD.

[22] Di Renzo L, Cioccoloni G, Falco S, Abenavoli L, Moia A, Sinibaldi Salimei P, De Lorenzo A (2018). Influence of FTO rs9939609 and Mediterranean diet on body composition and weight loss: a randomized clinical trial. J Transl Med.

[23] Hubacek JA, Pikhart H, Peasey A, Kubinova R, Bobak M (2011). FTO variant, energy intake, physical activity and basal metabolic rate in Caucasians. The HAPIEE study. Physiol Res.

[24] Hewagalamulage SD, Lee TK, Clarke IJ, Henry BA (2016). Stress, cortisol, and obesity: a role for cortisol responsiveness in identifying individuals prone to obesity. Domest Anim Endocrinol.

